# Prediction of *β*-thalassemia carrier using federated learning and explainable AI

**DOI:** 10.3389/fmed.2026.1687773

**Published:** 2026-01-30

**Authors:** Hafiz Ali Younas, Bilal Shoaib Khan, Abdul Hannan Khan, Anas Bilal, Asaad Algarni, Raheem Sarwar, Seyed Jalaleddin Mousavirad

**Affiliations:** 1 Department of Computer Science, Green International University, Lahore, Pakistan; 2 Department of Computer Science and IT, The University of Lahore, Lahore, Punjab, Pakistan; 3 College of Information Science and Technology, Hainan Normal University, Haikou, China; 4 Department of Computer Science, Faculty of Computing and Information Technology, King Abdulaziz University, Rabigh, Saudi Arabia; 5 OTEHM, Manchester Metropolitan University, Manchester, United Kingdom; 6 Department of Computer and Electrical Engineering, MidSweden University, Sundsvall, Sweden

**Keywords:** explainable AI, federated learning, LIME, SVM, *β*-thalassemia carrier

## Abstract

Millions of people worldwide suffer from *β*-thalassemia, an inherited blood disorder that requires precise carrier screening to avoid serious health issues. Conventional centralized screening techniques rely on combining patient data, which raises privacy and legal issues under regulations like General Data Protection Regulation (GDPR) and the Health Insurance Portability and Accountability Act (HIPAA). Although machine learning has increased the accuracy of diagnoses, its reliance on shared data raises issues with data security and makes it more difficult to develop collaborative models. Federated learning provides a solution by allowing multi-center collaboration and protecting privacy by training models locally at each clinical site and sharing only model parameters. In this work, a federated multi-kernel support vector machine (SVM) framework is developed, which aggregates updates through federated averaging and deploys linear, polynomial, radial basis function, and Deep kernel (DK) on client devices. We also incorporate explainable AI methods SHapley Additive exPlanations (SHAP) and Local Interpretable Model-agnostic Explanations (LIME) to decipher forecasts and pinpoint important hematological characteristics. Our federated model performs on par with centralized methods, achieving 98.4% accuracy, 99.2% sensitivity, and 98.8% specificity when tested on 5,066 complete blood count records. The most significant predictors, according to the explainability analyses, are hemoglobin level and mean corpuscular volume. Our results open the door for scalable, transparent, and compliant *β*-thalassemia screening across dispersed healthcare systems by demonstrating that federated multi-kernel SVMs with Explainable Artificial Intelligence (XAI) can provide high diagnostic performance while protecting patient privacy.

## 1 Introduction

Thalassemia is blood related disease, derived from two Greek words, i.e., “Thalassa” means sea and “Hema” means blood. In 1936, it was first identified by George Whipple (1). It is a widespread disease affecting most of the population globally, specifically Mediterranean, Africa, the Middle East, Asia, and Southeast Asia (2, 3). Annually, around 68,000 newborns are diagnosed with various types of Thalassemia, with approximately 80–90 million people being subjected to *β*-Thalassemia, which makes it 1.5% of the total world’s population. It has been revealed by the reports from the World Health Organization (WHO) that about 5.1% of the global population carries the *β*-Thalassemia gene. Moreover, recent research has depicted that over 350 mutations are linked to β-Thalassemia, out of which only 20 account for over 80% of these caused by geographical clustering that influences mutation distributions with migration patterns (4). Another prevalent disease that is affecting people worldwide is Anemia, closely associated with Thalassemia. Anemia is defined as hemoglobin levels below 12.0 g/dL in women and 13.0 g/dL in men, as defined by WHO, with factors, i.e., sex, ethnicity, and physiological status, necessitating more refined guidelines. Multiple causes for anemia require doctors to assess hematologic parameters, health conditions, and patient history for accurate diagnosis (5). Thalassemia results in reduced hemoglobin production that leads to severe anemia as mutations affect the hemoglobin structure, primarily alpha or beta globin chains, thereby disrupting their function and further complicating the health condition (6). Thalassemia is classified based on its severity (trait, carrier, intermediate, major) and affected hemoglobin components (alpha or beta). In addition, the structure of hemoglobin is composed of two alpha and two beta chains that are crucial for oxygen transportation. Thus, deficiency in either component may lead to alpha or beta Thalassemia, respectively (7). In Pakistan, approximately 9.8 million individuals are carriers of *β*-Thalassemia. Thus, early detection is necessary. The high prevalence of carriers is a key concern, as there is a 25% probability of a child inheriting the disease if both parents are carriers. Compounding this, many carriers are unaware of their status due to negligence. However, timely screening could prevent severe health complications and fatalities (8). Diagnosing and distinguishing Thalassemia from other Hemoglobin (Hb) diseases is currently challenging because clinical data, including blood smear images can be unreliable. Moreover, the traditional screening techniques, for example, Deoxyribonucleic acid (DNA) testing, high-performance liquid chromatography (HPLC), hemoglobin electrophoresis, and Polymerase Chain Reaction (PCR) mutation analysis, are proven to be very effective but costly that requiring specialized equipment, making accessibility difficult in an area with limited resources (9, 10).

However, these challenges are overcome by the advancements in machine learning (ML) in various domains, including medical diagnostics, autonomous systems, and language processing. ML operates on self-improving algorithms that are capable of analyzing large datasets, recognizing complex patterns, and optimizing predictive accuracy. These algorithms model refined data distributions using statistical and probabilistic approaches, making them effective in the medical sector. It has been found that ML-based diagnostic approaches have significant applications in the medical field, such as diagnosing brain tumors, lung disorders, and iron deficiency anemia (11–14). Several AI based models have been implemented for the improvement of disease detection, including K-nearest neighbors (KNN) (15–17), deep neural networks (DNN) (18, 19), and support vector machines (SVM) (20, 21). Moreover, to combat the widespread prevalence of thalassemia, it is important to adopt advanced diagnostic systems instead of relying on conventional screening. The necessity for low-cost, accessible, and efficient screening solutions becomes challenging, especially in underprivileged regions. This study aims to establish a cost-effective and accurate method for detecting *β*-Thalassemia carriers using machine learning techniques on red blood cell indices obtained from complete blood count (CBC) tests. The study leverages a dataset of 5,066 patient blood tests and Federated Learning to improve model performance while keeping data decentralized. In parallel, tightening federal (HIPAA) and emerging state-level health privacy statutes (e.g., New York and California) are driving the integration of privacy-preserving and AI-based monitoring mechanisms to ensure regulatory compliance in medical AI systems (22).

This study introduces Federated Learning for *β*-thalassemia carrier detection to enable collaborative model training on distributed CBC datasets while avoiding the centralization of raw patient data, thereby mitigating HIPAA/GDPR-related privacy and security risks. By training multi-kernel SVMs across heterogeneous clinical sites and aggregating models rather than data, the framework aims to achieve high diagnostic accuracy, robust generalization, and regulatory-compliant, privacy-preserving large-scale screening of *β*-thalassemia carriers.

The proposed study integrates Federated Learning with machine learning algorithms, including Support Vector Machine (SVM) with multiple kernel variations, to develop a highly accurate *β*-Thalassemia screening model. This research introduces a novel method that ensures high accuracy as compared to previous approaches that relied on preprocessing techniques. This model is constructed to classify individuals as *β*-Thalassemia carriers with greater precision by training three machine learning models. The proposed method is reliable and affordable that providing an effective solution for early detection and prevention of the disease. Moreover, this paper is structured with Section 2 reviewing existing literature, Section 3 describing the method and material, Section 4 presenting the proposed framework, Section 5 outlining the results and discussion, and Section 6 concluding the article. The focus of this study is to provide an innovative and privacy-preserving framework for the detection of *β*-Thalassemia by utilizing Federated Learning to improve public health outcomes.

## 2 Related work

Detecting a thalassemia carrier is challenging as the symptoms are not very obvious, also medical expertise and detailed patient data are required for its identification, which makes it is difficult and at the same time expensive to assess the early detection. Thus, an automated system for predicting thalassemia carriers is crucial to prevent this disease from spreading to future generations in future. The effectiveness of machine learning models has various applications involving a significant importance of these models in carrier prediction for thalassemia patients (20). In one of the recent studies conducted in 2024 by Uçucu and Azik, a machine learning model to differentiate between iron deficiency anemia (IDA) and *β*-thalassemia minor (BTM) using only complete blood count (CBC) data was introduced. It was found that artificial neural networks (ANNs) outperformed by offering a rapid and cost-effective alternative to conventional methods. Thus, by the incorporation of AI, healthcare providers can more efficiently and accurately distinguish between IDA and BTM, which leads them to an improvement in patient care (23). Recent advancements in thalassemia treatments are transforming patient care. For example, in North America, these new techniques are utilized for measuring tissue iron and innovative therapies for iron removal. Life expectancy has been increased by these medical advancements, but with these survival rates, new challenges are rising, including endocrinopathies and hepatitis, significantly impacting quality of life (24). In light of these challenges, machine learning and federated learning offer substantial potential for enhancing the diagnostic process of thalassemia. Federated learning, in particular, is a promising approach, allowing multi-center collaborations while ensuring patient data security by training models locally and sharing only model parameters. This method addresses concerns related to centralized data aggregation, which often raise issues regarding data privacy under regulations like GDPR and HIPAA. Recent studies have demonstrated the effectiveness of machine learning techniques, such as multi-kernel support vector machines (SVMs), in diagnosing *β*-thalassemia, incorporating explainable AI (XAI) methods like SHAP and LIME to provide transparency and highlight key diagnostic features such as hemoglobin levels and mean corpuscular volume (25–27). Recent studies have demonstrated the effectiveness of machine learning techniques, such as multi-kernel support vector machines (SVMs), in diagnosing *β*-thalassemia, incorporating explainable AI (XAI) methods like SHAP and LIME to provide transparency and highlight key diagnostic features such as hemoglobin levels and mean corpuscular volume (25–27).

Incorporating federated learning with SVMs can significantly improve diagnostic performance across decentralized healthcare systems without compromising data privacy. For instance, federated multi-kernel SVM models have shown high diagnostic accuracy and sensitivity when tested on large datasets, making them ideal for large-scale *β*-thalassemia screening programs (28–30). Moreover, research into gene therapies and disease prediction models underscores the potential of combining AI with medical treatments, opening a path for more personalized care (31, 32). Furthermore, the integration of AI into medical diagnostics aligns with global regulatory frameworks, ensuring compliance with privacy standards while enhancing diagnostic capabilities (33, 34). The ongoing development of AI models, including advancements in protein localization prediction and gene-disease relationships, is expected to further enhance the effectiveness of AI-based diagnostics for *β*-thalassemia and other genetic disorders (35–38).

The study entitled “Identifying *β*-thalassemia carriers using a data mining approach: The case of the Gaza Strip, Palestine” emphasized the significance for the identification of identifying β-thalassemia carriers as a critical factor in effective genetic counseling and disease management, particularly in highly prevalent areas. The research employed data mining techniques and machine learning algorithms to analyze a dataset that contains genetic and hematological information of the Gaza Strip individuals. The results indicated that carriers can be effectively identified with these techniques to improve early detection. The application of data mining in a healthcare context in this paper introduces a novel approach that can enhance the screening programs in high-risk areas. However, this study is limited in generalizability because of the dataset’s demographic and geographic specificity (15). In 2023, Tran et al. explored the prevalence of thalassemia in Vietnam and developed a clinical decision support system (CDSS) for prenatal screening involving the analysis of over 10,000 medical records of first-time pregnant women and their partners. Four AI-based CDSSs, training machine learning models on 1,992 cases, were designed and evaluated with 1,555 cases. These AI-based systems demonstrated high accuracy in identifying thalassemia carriers, thereby suggesting AI for enhancing prenatal screening processes (39). Similarly, a study by Das et al. presented a valuable decision support scheme for beta thalassemia and HbE carriers that was effective, but while addressing data privacy, ethical implications are necessary for protecting sensitive genetic information (40). In Ferih et al. (41), researchers investigated AI-driven enhancements in thalassemia diagnosis with a review of machine learning and deep learning techniques used on clinical datasets. The results proved the effectiveness of AI in enhancing diagnostic accuracy and personalized treatment. This research’s limitations include dataset variability and broader validation for diverse populations (41).

The application of artificial intelligence and machine learning techniques has improved the identification of thalassemia gene carriers, specifically in non-anemic populations, which are often overlooked by conventional screening methods. Machine learning models are more trained on blood sample datasets, thereby ensuring higher accuracy in detecting carriers in comparison to traditional approaches. These models produce efficient results by the analysis of erythrocyte morphology and hematological parameters, i.e., hemoglobin levels, mean corpuscular volume, and red blood cell count. Many studies underscore the potential of deep learning, i.e., deep neural networks, in the early detection of thalassemia with greater prediction accuracy. However, the size and diversity of datasets remain a challenging factor for greater scalability in this research. As the AI-driven methods offer greater screening efficiency and more benefits to public health, these models also require further validation with larger and diverse datasets. This issue has been addressed by recent approaches that overcome the challenges found in identifying thalassemia gene carriers for individuals with no anemia, as all the characteristics of red blood cells can be analyzed via these innovations. For instance, research applied a deep neural network on indices of red blood cells, including hemoglobin level, mean corpuscular volume, and cell count. The data was sourced from thalassemia patients and healthy controls, where AI-based model results in improved carrier detection accuracy. These models examined erythrocyte morphology with index data, thereby surpassing conventional methods and indicating a more efficient screening method. However, the success of this methodological framework has a limitation with limited sample diversity and potential algorithmic biases that can alter the results (42).

Another article with the title as “Role of Red Cell Indices in Screening for Beta Thalassemia Trait: An Assessment of the Individual Indices and Application of Machine Learning Algorithm” utilized a dataset with red cell indices having parameters, i.e., mean corpuscular volume (MCV), mean corpuscular hemoglobin (MCH), and red blood cell count from beta thalassemia patients. In this research, machine learning algorithms are incorporated to improve screening accuracy. However, dataset details are not discussed in this article, which potentially affects the generalizability of results (43). Similarly, Bharath et al. (44) analyzed a dataset containing clinical and laboratory data from patients with alpha thalassemia and healthy individuals. The study demonstrated the effectiveness of machine learning classifiers to improve the screening accuracy. This research also utilized explainable AI techniques to interpret results with a greater understanding. Limitations still exist with details on dataset size and diversity (44). Another study classifies beta-thalassemia major and HbE/beta-thalassemia with the aid of deep learning techniques by applying them to image structure function images for accurate classification. The results demonstrated that the proposed study has achieved a higher accuracy, thereby showcasing the potential for deep learning in hematological diagnostics with improved precision and automated classification. The limitations include the need for extensive datasets across diverse populations (45).

Compliance measures are necessary for the Privacy concerns of AI in medical applications. Moore and Frye reviewed HIPAA’s historical background, patient health information (PHI), and privacy and security rules regarding healthcare data, emphasizing ongoing compliance challenges. The review depicted the critical role of HIPAA in protecting patient privacy and security (46). In the study (47), the authors analyzed the intersection of AI technologies and the HIPAA Privacy Rule, which focused on AI applications’ navigation in the health sector for patient privacy regulations. The article also discussed a few challenges while integrating AI into healthcare practices, along with guaranteed compliance with HIPAA standards. As digital dependence is rising, extensive data growth continues to increase with global privacy laws becoming imperative. For this purpose, Bakare et al. compared the EU’s General Data Protection Regulation (GDPR) with the U. S. privacy frameworks and analyzed all compliance challenges across jurisdictions (48). Moreover, ethical and regulatory concerns are also rising in AI integration for radiology. Pesapane et al. analyzed these challenges of AI as medical devices in the EU and U. S. frameworks and highlighted ethical considerations and regulatory landscapes that shape the AI deployment in healthcare (49). Similarly, Mennella et al. also explored the ethical and regulatory complexities of AI in healthcare and emphasized the frameworks that include AI adoption in clinical settings (50).

Ensemble learning reduces errors and manages the bias-variance trade-off to enhance robustness against noise and identify intricate data patterns by voting or weighted averaging the predictions of numerous base models. Because ensembles use different learners, they typically perform better than models in high-dimensional or class-imbalanced environments. By reducing errors and maintaining model strengths, these strategies transform weak learners into strong ones. In sensitive or remote settings, traditional ensemble systems require centralized access to all training data, which can present logistical and privacy challenges. Emerging federated and distributed ensemble frameworks balance performance gains with data confidentiality requirements by enabling collaborative training without sharing raw data, thereby overcoming these limitations. Recent developments in the classification of cancer from microarray data have demonstrated the effectiveness of hybrid feature selection techniques, which combine metaheuristic algorithms like Particle Swarm Optimization (PSO), Cuckoo Search, and Artificial Bee Colony (ABC) with filter techniques like minimum redundancy maximum relevance (mRMR). Through ensemble learning, Panigrati et al. showed how combining these techniques with classifiers such as Support Vector Machines (SVM), Random Forests, and Multilayer Perceptron (MLP) improves accuracy and lessens single-model bias. Sahu et al. built on this by using a two-stage mRMR–Binary Portia Spider Optimisation Algorithm (BPSOA) pipeline, which significantly reduced false-negative and misclassification rates while achieving peak accuracies of up to 99.79% across six cancer microarray datasets, with F1-scores exceeding 99% and Matthews Correlation Coefficients (MCCs) exceeding 96% (51, 52).

Sahoo et al., Despite targeted therapies, between 30 and 40 percent of patients with HER2-positive breast cancer experience metastases or relapse. They trained six base classifiers (SVM, logistic regression, decision tree, random forest, AdaBoost, and XGBoost) using TCGA data from 123 HER2-positive cases. They then combined the results using ensemble weighted averaging, soft voting, and hard voting. With the best performance, the weighted-averaging ensemble had 88.46% accuracy, 89.74% precision, 94.59% sensitivity, 73.33% specificity, 92.11% *F*-value, a Matthews correlation coefficient of 0.7107, and AUC = 0.903. This shows that combining H&E histopathology with clinical data can accurately predict metastasis and relapse, leading to better treatment decisions (53). Pand et al. describe that globally, the incidence of cancer is on the rise. Machine learning (ML) applied to high-dimensional microarray data can facilitate this screening, but without efficient feature selection, performance deteriorates. Therefore, we propose BIMSSA, a pipelined feature-selection framework that optimizes feature size using the Swarm Optimisation Algorithm (SSA) after applying Boruta and Improved Maximum Relevance Minimum Redundancy (IMRMR) to isolate relevant gene subsets. As base learners, five classifiers are used: SVM, Random Forest, Extreme Learning Machine, AdaBoost, and XGBoost. The top three are chosen by majority vote to create the final ensemble. Tested on four cancer microarray cohorts (ALL-AML, Lymphoma, MLL, and SRBCT), BIMSSA demonstrated a strong ability to predict a variety of cancer types and provided a potent diagnostic tool for researchers and clinicians with accuracies of 96.7, 96.2, 95.1, and 97.1%, respectively (54).

A framework to guarantee the confidentiality and security of patient datasets has emerged, which is privacy-preserving machine learning (PPML). Various advanced algorithms have been developed to address these concerns, while federated learning technique stands out as it enables model training across diversely distributed data sources without centralizing raw data. Differential privacy further has enhanced the security by controlling noise in datasets and preserving the privacy of all individuals. Other notable techniques that anonymize predictions through ensemble models include Private Aggregation of Teacher Ensembles (PATE). Moreover, Secure Multi-Party Computation (SMPC) allows collaborative computation without exposure to the data. De-identification processes are also important to strip datasets of personally identifiable information. In our study, federated learning is prioritized as it has greater scalability, ability to preserve data locality, and efficiency, which makes it suitable for healthcare applications.

Li et al. characterized federated learning as a decentralized machine learning that can facilitate collaborative model training across multiple entities without the exchange of data, as it is critical in privacy-sensitive sectors including healthcare, finance, and telecommunications (55). Furthermore, federated learning applications can enhance data security and improve model adaptability, thereby reducing operational costs, aligning with regulatory standards, i.e., GDPR. Both of the sources have emphasized their dual role to reduce privacy risks with diverse data resources, underscoring the significance of enabling secure and collaborative learning without compromising sensitive information (56). Shen et al. explored the transformation of distributed machine learning to federated learning and emphasized its role in addressing privacy concerns in data. It also described federated learning as a decentralized mechanism enabling multiple participants to collaboratively train machine learning models without sharing any raw data while proving its significance in healthcare, finance, and telecommunications. The research emphasized that federated learning not only alleviates privacy concerns by maintaining data locality but also enhances model generalization with diverse datasets. However, the risks for potential privacy, such as inferring sensitive information, persist with model updates (57).

The Contribution of the proposed work is the creation of a sophisticated, privacy-preserving federated learning framework specifically designed for the early identification of thalassemia carriers. The proposed model represents a significant advancement in medical diagnosis and privacy-focused AI applications, decentralized training across multiple data sources without compromising sensitive patient information.

The key contributions and achievements of this model as:

- Proposed federated learning architecture ensures the privacy of patients’ data is maintained accordingly to international laws like HIPAA/GDPR-compliant privacy. It allows several clinical sites to work together to train SVM models on local CBC data without exchanging raw patient records.
- Multi-Kernel SVM Optimization at the Client Side: We apply and contrast three SVM kernels (Linear, Polynomial, and RBF) on decentralized data, showing how each identifies unique hematological patterns and offers supplementary information.
- Proposed Global Model Aggregation attain 98.4% accuracy and strong generalization across diverse patient demographics by combining client-side kernel models into a global RBF-based classifier.
- Explainable AI Integration (SHAP and LIME): To promote openness and clinical trust, we use SHAP summary plots and LIME local explanations to clarify feature significance and individual predictions.

## 3 Methodology

This section discusses the details of datasets, preprocessing techniques, and machine learning utilized in developing a predictive model for the identification of *β*-thalassemia carriers to ensure a rigorous training and validation process.

### 3.1 Dataset description

The dataset employed in this research was gained from the Punjab Thalassemia Prevention Programme PTPP, which is a reliable and comprehensive source of thalassemia records (21, 58). PTPP conducts screening of over 300,000 patients annually for identifying thalassemia carriers and also provides medical care to thalassemia major patients. This project prioritizes screening in extended family members to eliminate any risk of carrier prevalence, thereby emphasizing the strategies for preventive measures. It also recommends evaluations when someone in the family is diagnosed with thalassemia to ensure early detection.

**Figure 1:**
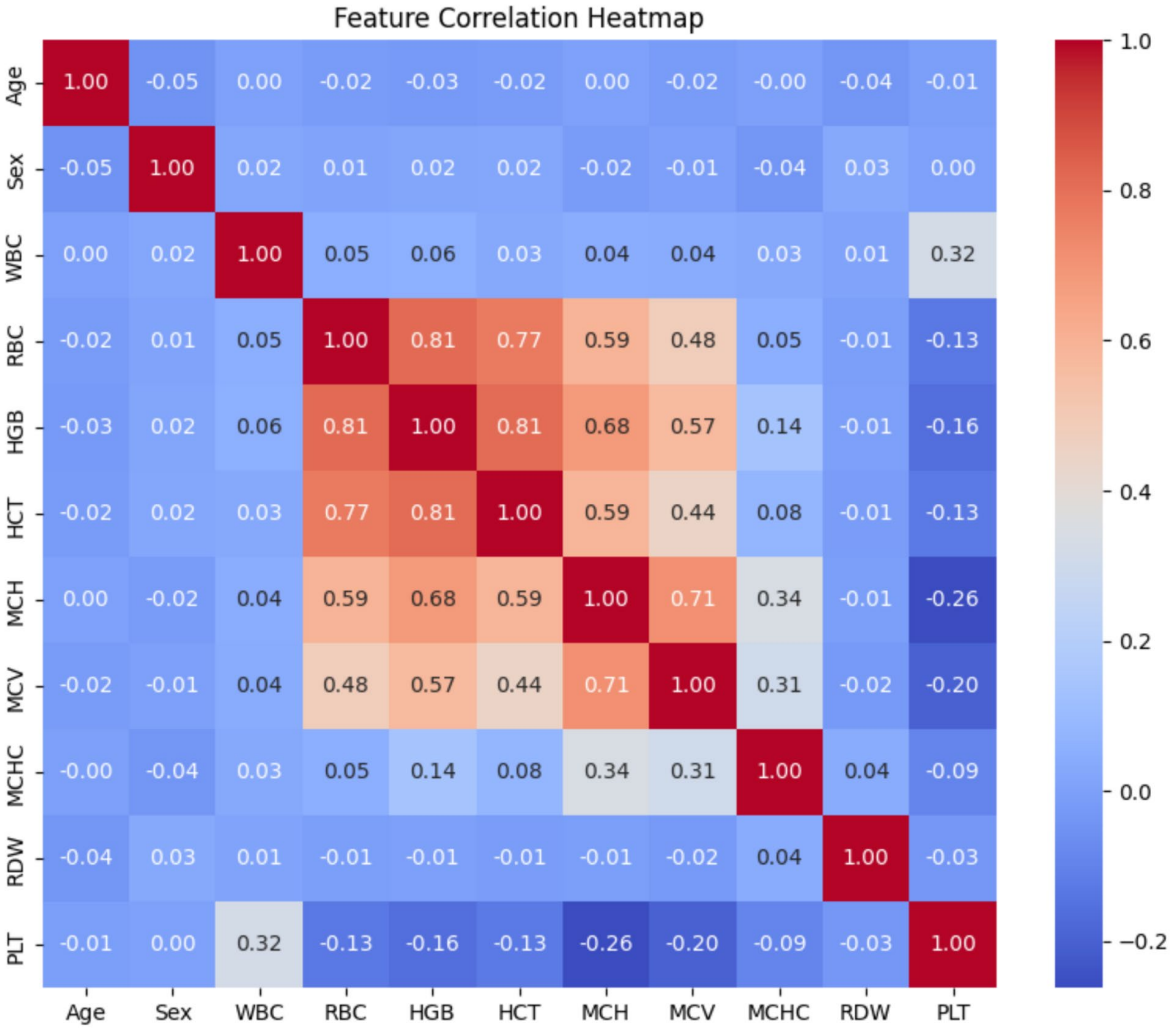
Feature correlation heatmap.

The attributes of the dataset used in this research are shown in Figure 1. It contains the data of 5,066 patients, with 3,051 classified as non-carriers and 2,015 identified as carriers of thalassemia in 2024. The categories of the data are based on gender and age, where 53% of the patients were male and 47% were female. Moreover, it shows that 54% were adults and 46% were children.

### 3.2 Features of the dataset

Combination of complete blood count (CBC) parameters is incorporated as the key features in this study where CBC parameters include red blood cell count (RBC), white blood cell count (WBC), hematocrit (HCT), red cell distribution width (RDW), mean corpuscular volume (MCV), mean corpuscular hemoglobin (MCH), mean corpuscular hemoglobin concentration (MCHC), platelet count (PLT) and hemoglobin levels (HB) and further required details are also mentioned in Table 1. It also includes demographic factors, i.e., age and gender.

**Table 1:** Description of dataset.

Feature	Value
Dataset name	β-thalassemia carrier
Total instances	5,066
Total feature	11
Class	0 (non-carrier) and 1 (carrier)
β-thalassemia carrier	2,594
β-thalassemia non-carrier	2,472
Data type	Numerical

### 3.3 Data preprocessing

The data was preprocessed to remove any inconsistencies or errors, as in this way the data has a significant impact on results. To certify clean data, missing records and an unrated. The healthiness system of measurement was eradicated. Non-essential identifiers such as the name of the patients, interaction details, and test dates were also removed. The data are classifying patient ages obsessed by two groups: children (0–18 years) and grown persons (above 18 years), characterized by binary values 0 and 1, correspondingly, and biological genders were prearranged with female as 0 and male as 1. Further, the target class is also represented as 0 and 1; *β*-thalassemia carriers were categorized as 1 and non-carriers as 0. This preprocessing approach minimized the noise and enhanced data readiness, and well-looked-after analytical consistency, as long as a robust foundation for model training and assessment.

### 3.4 Exploratory data analysis

**Figure 2:**
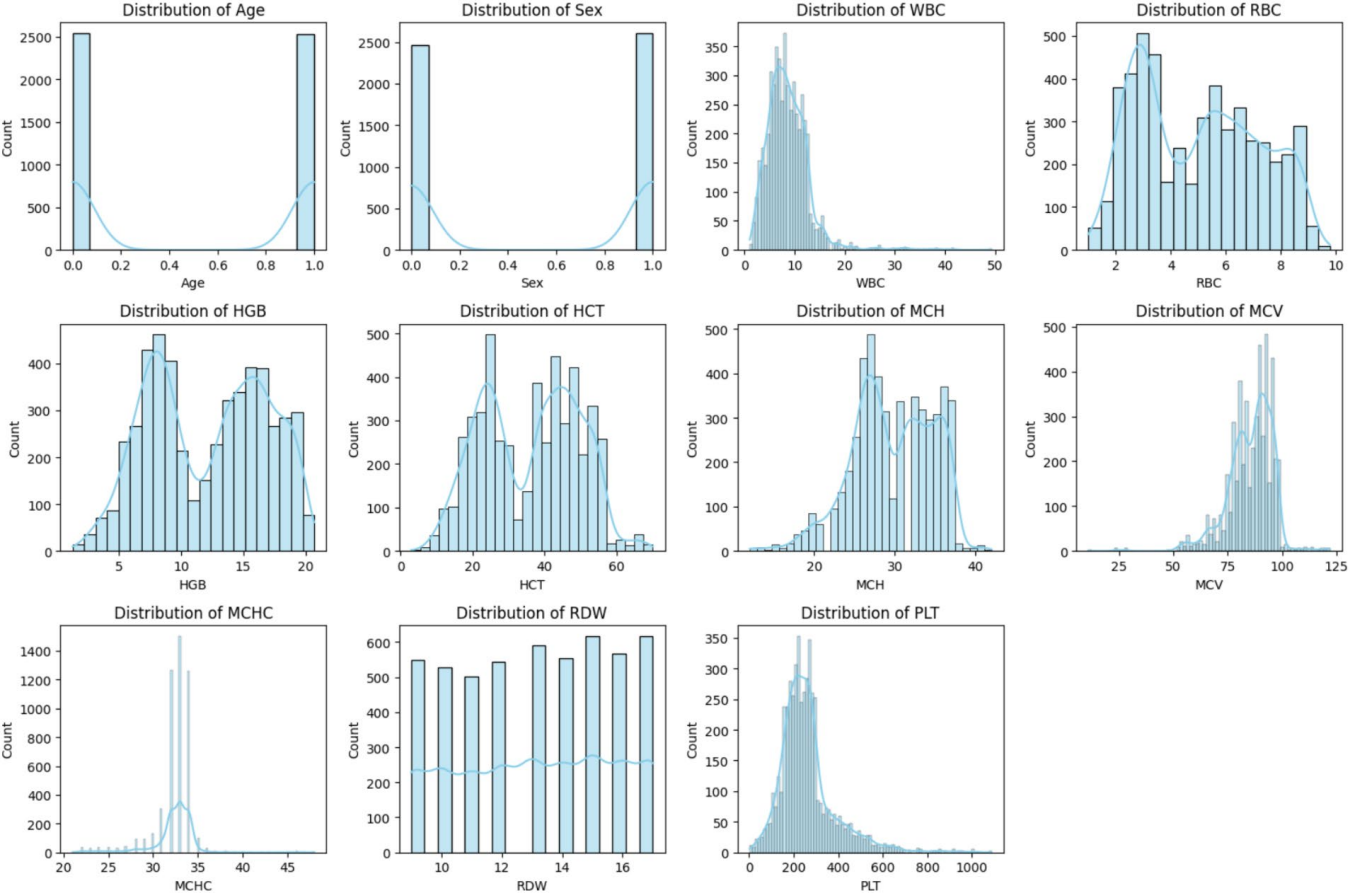
Histogram of each feature.

**Figure 3:**
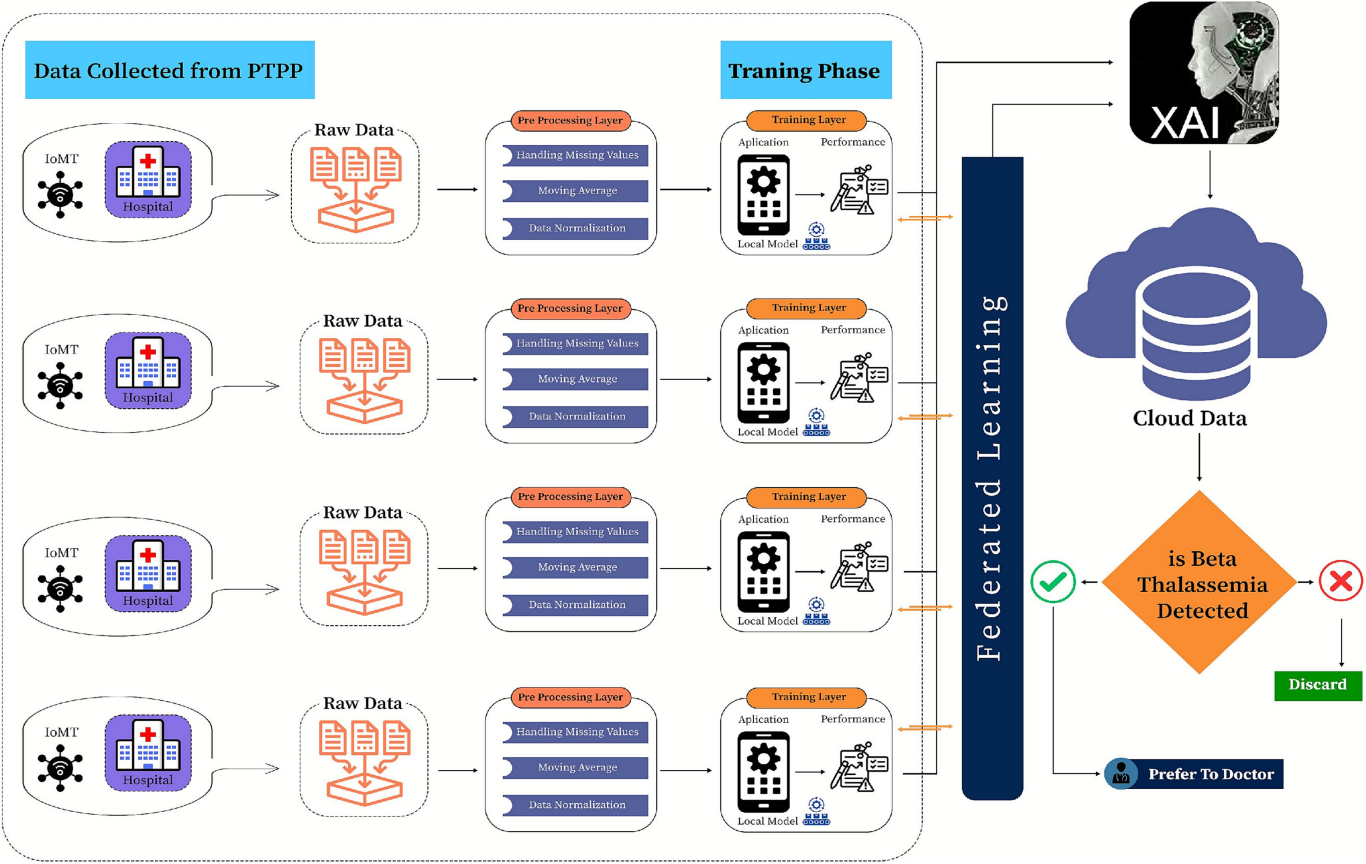
Proposed federated learning-based model for β-thalassemia carriers identification.

To better understand the relationship of different features with each other, various graphical techniques are used. These visuals help in identifying the trends, clusters, and unusual data points in the dataset. A correlation heatmap is utilized in the proposed research as depicted in Figure 1. For the analysis of features, Correlation Heatmap, the histograms are shown in Figure 2, and the boxplots are shown in Figure 3. These figures illustrate the identification of correlations between variables and detecting potential outliers, and offer a more comprehensive understanding of the dataset.

Figure 2 shows the overview of the feature values ‘distribution with data points spread across different ranges. Most of the observations accumulate within a central range and some of the observations extend toward the higher values to form a right-skewed distribution. Potential outliners are indicated by a few distant bars on the extreme right. Thus, for exploratory data analysis, histograms are considered as a fundamental component as they enhance the reliability and accuracy of the prepared ML models.

## 4 Proposed framework

This study introduces a framework for classifying *β*-thalassemia carriers (Jane classification) by integrating clinical biomarkers with a multi-kernel Support Vector Machine (SVM) architecture. The methodological workflow is structured into a systematic three-phase design to ensure accuracy and efficiency in classification, as shown in Figure 3.

Exploratory analysis involves data profiling and visualization to identify trends, outliers, and correlations among features.

Preprocessing: applies robust normalization and imputation techniques to address inconsistencies such as missing values and skewed distributions.

Model development: benchmarks four SVM kernels (linear, radial basis function, polynomial, and Deep Kernel) to capture both linear and nonlinear feature interactions, with hyperparameter optimization performed using grid search and 10-fold cross-validation to enhance model accuracy and performance.

To tackle scalability and privacy challenges in clinical data sharing, this framework adopts a federated learning approach, where locally trained SVM models (linear, poly, and RBF kernels) at healthcare facilities periodically merge insights into a global polynomial kernel model. This collaborative method reduces raw data exchange while improving diagnostic generalizability across diverse populations. Model performance is systematically assessed using accuracy, precision, recall, and F1 scores, and specificity, with SHAP values providing interpretability by identifying fundamental biomarkers. The incorporation of federated learning ensures compliance with GDPR while facilitating practical implementation in resource-constrained clinical environments.

### 4.1 Model development using SVM with multiple kernels

Support Vector Machines (SVMs) are perfect for *β*-thalassemia carrier detection because they maximize the margin between classes, which optimizes the decision boundary and encourages strong generalization even when there are a lot of features compared to the sample size, as in our case. This study also employed SVMs with three distinct kernel functions: Linear, Radial Basis Function (RBF), and Polynomial for recognizing specific data patterns. The efficiency of these kernels is assessed individually at the client side, which guarantees evaluation of their classification performance. Cross-validation is utilized to validate generalization capabilities, and performance metrics such as accuracy, F1-score, and confusion matrices are analyzed. Moreover, diverse analytical techniques, i.e., Receiver Operating Characteristic (ROC) curves, Principal Component Analysis (PCA) based decision boundary visualizations, and learning curve analyses, were applied for the enhancement in the model’s interpretability and comparison using three different kernels.

The line Equation can be expressed as

$P_{0}=qP_{1}+\mathrm{sq}$ is the slope and s is the intercept. $P_{0}$ is the output given the input


$$qP_{1}-P_{0}+s=0$$ (1)

Let $\vec{P}={\left(P_{0},P_{1}\right)}^{T}$ and $\vec{r}={\left(r_{1},r_{2}\right)}^{T}$ , and $r_{1}=q,r_{2}=-1$ then Equation 1. Can be written as


$$\vec{r.}\vec{P}=0$$ (2)

Equation 2 is obtained from the vectors in two dimensions only, and this equation is also valid for higher dimensions.

The direction the vector $\vec{P}={\left(P_{0},P_{1}\right)}^{T}$ is defined in Equation 3 as


$$\vec{P}=\frac{p_{0}}{\left\|p\right\|}+\frac{p_{1}}{\left\|p\right\|}$$ (3)

Where p is the magnitude of vector P and is written for the double vectors detailed in Equation 4 as


$$\|p\|=\sqrt{p_{0}^{2}+p_{1}^{2}}$$ (4)

For n vectors, Equation 4, may also be rewritten as:


$$p=\sqrt{p_{0}^{2}+p_{1}^{2}+p_{2}^{2}+p_{3}^{2}+\ldots +p_{n}^{2}}$$

And in terms of angle, it will be written in Equation 5 as:


$$\cos\theta =\frac{p_{0}}{\left\|p\right\|}\;and\;\cos\omega =\frac{p_{1}}{\left\|p\right\|}$$ (5)

By putting the values of Equation 5 into Equation 5 and will be rewritten as:


b=(cosθ,cosω)

$$\vec{r}.\vec{p}=\|r\|\;\;\|p\|\cos\theta$$

Where θ=η−ω , so cosθ=cos(η−ω) , By applying the formula cos(x−y)=cosxcosy+sinxsiny , we get.

cos(θ)=cosηcosω+sinηsinω . By applying this in Equation 5 we have


$$\cos\theta =\frac{r_{1}}{\left\|r\right\|}\frac{p_{0}}{\left\|p\right\|}+\frac{r_{2}}{\left\|r\right\|}\frac{p_{1}}{\left\|p\right\|}$$

$$\cos\theta =\frac{r_{1}p_{1}+r_{2}p_{2}}{\left\|r\|\;\;\|p\right\|}$$

After cancelation the above equation will be written as


$$\vec{r}.\vec{p}=\sum_{k=1}^{n\;}r_{k}p_{k}$$

Let u=v(r.p+s)

Here u is the functional margin of the dataset and is defined as


$$u=\min_{k=1,2,3,4,\ldots n}u_{k}$$

$$u_{k}=v_{k}(r.p+s)$$

The hyperplane with the largest u will then be further chosen that depicts the margin of the dataset geometrically. Once the primary objective of finding the optimal hyperplane has been achieved then it is easy to find out the corresponding values of r and s. The Lagrange function is expressed in Equation 6.


$$\min L=\frac{1}{2}\|w\|;g(x):y_{i}(wx_{i}+s)-1=0$$

$$\min_{L}f(r,,s,,q)=\frac{1}{2}r^{2}-\sum a_{i}[y_{i}(r.p+s)-1]\;w.r.\text{to}\;r,b$$ (6)

By expanding Equation 6 we get


$$\min L=\frac{1}{2}r^{2}-\sum a_{i}y_{i}(r.p+s)+\sum a_{i}\;\;w.r.\text{to}\;w,s$$

So the Lagrange problem of SVM with L regularization is depicted in Equation 7, as


$$\min L_{p,s.v.m}=\frac{1}{2}r^{2}-\sum_{i=1}^{l}a_{i}y_{i}(r.p+s)+\sum_{i=1}^{l}a_{i}\;\;\;\forall_{i},a_{i}\geq 0$$ (7)

Where l is the number of training data points and while taking the derivative w and s the Lagrange in Equation 7 we get


$$\frac{\delta L_{p,s.v.m}}{\mathrm{\delta w}}=r-\sum_{i=1}^{l}a_{i}y_{i}p_{i}=0$$

$$\frac{\delta L_{p,s.v.m}}{\mathrm{\delta b}}=\sum_{i=1}^{l}a_{i}y_{i}=0$$

So


$$r=\sum_{i=1}^{l}a_{i}y_{i}p_{i}\&\sum_{i=1}^{l}a_{i}y_{i=0}\;$$ (8)

Now we have to solve this optimization problem for the dual of the original problem. In the dual problem instead of minimizing over w, b subject to the constraints involves a’s. we can minimize over a (the dual variable) also subject to the same variables relations that are obtained previously for r as in Equation 8, and s. The minimization of the loss function over a is given in Equation 9, as


$$\max L_{D}(a_{i})=\sum_{i=1}^{L}a_{i}-\frac{1}{2}\sum_{i=1}^{l}a_{i}a_{j}y_{i}y_{j}(P_{i}P_{j})\;\;S.t\;\sum_{i=1}^{l}a_{i}y_{i}=0\&a_{i}\geq 0\;$$ (9)

The Lagrange multiplier for the S. V. M can be further extended to the Kuhn-tucker method. And will be explained as


$$a_{i}[y_{i}(r_{i}.P^{*}+s)-1]=0$$

Here $P^{*}\;$ is the optimal point and the term $a_{i}$ shows the positive behavior. So, we have


$$y_{i}[(r_{i}.P^{*}+s)-1]=0$$ (10)

Equation10, depicted the closest support vectors to the hyperplane


$$r-\sum_{i=1}^{l}a_{i}y_{i}P_{i}=0$$

$$r=\sum_{i=1}^{l}a_{i}y_{i}P_{i}$$ (11)

The value of r in terms of y is detailed in Equation 11, To compute for the value of y, we have


$$y_{i}((r_{i}.P^{*}+s)-1)=0$$

Multiply $y_{i}$ on both sides we get and after simplification the final form is given in Equation 12.


$${y_{i}}^{2}((r_{i}.P^{*}+s)-y_{i})=0$$

$$(r_{i}.P^{*}+s)-y_{i}=0$$

$$(r_{i}.P^{*}+s)=y_{i}$$ (12)

The hypothesis function represents the support vectors for the optimal hyperplane, is written in Equation 13 as


$$h(r_{i})=\{\begin{array}{c} 1\;\;\;\text{if}\;r.p+s\geq 0\\ 0\;\;\text{if}\;r.p+s<0\; \end{array}\}$$ (13)

#### 4.1.1 Client models

In this study, we evaluated three SVM kernel functions, Linear, Polynomial, and RBF, using a uniformly preprocessed, standardized, and class-balanced dataset.

##### 4.1.1.1 Linear

The Linear kernel, valued for its simplicity and its effectiveness in separating data with a linear decision boundary (59), yielded steady performance with aggregated metrics (precision, recall, and F1 score) averaging around 0.957, and a mean cross-validation accuracy of approximately 0.948.

Linear kernel function, Equation 14 written as


$$k(p,y)=P_{i}^{T}.y_{i}$$ (14)

Weight update using Linear kernel is detailed in Equation 15 as


$$W^{L}={\left[\begin{array}{ccc} W_{1x1}^{L} & W_{1x2}^{L} & W_{1x3}^{L}\cdots W_{1\mathrm{xm}}^{L}\\ W_{2x1}^{L} & W_{2x2}^{L} & W_{2x3}^{L}\;\ldots \;W_{2\mathrm{xm}}^{L}\\ \begin{array}{c} W_{3x1}^{L}\\ \vdots \\ W_{\mathrm{nx}1}^{L} \end{array} & \begin{array}{c} W_{3x2}^{L}\\ \vdots \\ W_{\mathrm{nx}2}^{L} \end{array} & \begin{array}{ccc} \begin{array}{c} W_{3x3}^{L}\\ \vdots \\ W_{\mathrm{nx}3}^{L} \end{array} & \begin{array}{c} \ldots \\ \ddots \\ \cdots \end{array} & \begin{array}{c} W_{3\mathrm{xm}}^{L}\\ \vdots \\ W_{\mathrm{nxm}}^{L} \end{array} \end{array} \end{array}\right]}_{\mathrm{NxM}}$$ (15)

##### 4.1.1.2 Polynomial

The Polynomial kernel, designed to capture higher-order feature interactions in data that lack clear linear separability (60), achieved slightly improved outcomes, with overall metrics reaching near 0.985 and a mean CV accuracy of about 0.988.

Describe the Poly kernel function in Equation 16 written as.


$$k(p,y)={\left(p.y+c\right)}^{d}$$ (16)

Equation 17 specifies the weight update using a Poly kernel.


$$W^{P}={\left[\begin{array}{ccc} W_{1x1}^{p} & W_{1x2}^{p} & \begin{array}{ccc} W_{1x3}^{p} & \cdots & W_{1\mathrm{xm}}^{p} \end{array}\\ W_{2x1}^{p} & W_{2x2}^{p} & \begin{array}{ccc} W_{2x3}^{p} & \ldots & W_{2\mathrm{xm}}^{p} \end{array}\\ \begin{array}{c} W_{3x1}^{p}\\ \vdots \\ W_{\mathrm{nx}1}^{p} \end{array} & \begin{array}{c} W_{3x2}^{p}\\ \vdots \\ W_{\mathrm{nx}2}^{P} \end{array} & \begin{array}{ccc} \begin{array}{c} W_{3x3}^{p}\\ \vdots \\ W_{\mathrm{nx}3}^{p} \end{array} & \begin{array}{c} \ldots \\ \ddots \\ \cdots \end{array} & \begin{array}{c} W_{3\mathrm{xm}}^{P}\\ \vdots \\ W_{\mathrm{nxm}}^{P} \end{array} \end{array} \end{array}\right]}_{\mathrm{NxM}}$$ (17)

##### 4.1.1.3 Radial basis function

Notably, the RBF kernel, renowned for mapping data into an infinite-dimensional space to uncover complex nonlinear patterns (61, 62) delivered the highest performance, with aggregated scores close to 0.989 and a mean cross-validation accuracy near 0.991. Table 2 encapsulates these fundamental results, clearly demonstrating the incremental benefits of each kernel.

**Table 2:** Limitations of the previous work discussed.

References	Year	Technique/machine learning models	Kernal function	Feature selection method	Dataset	Accuracy	FL	XAI
AlAgha et al. (15)	2018	k-NN, NB, DT, and MLP.	–	–	44,360	99.5	✗	✗
Patgiri and Ganguly (16)	2021	k-NN, and NB.	–	CSF		98.87	✗	✗
Paokanta et al. (17)	2010	(BNs), MLR, KNN, MLP, and NB.	–	–	124	88.98	✗	✗
Das et al. (20)	2022	ANN, CART, CHAID, CRUISE, DT, E-CHAID, ELM, GUIDE, KNN, LR, MDA, MLA, MLP, NB, PLS-DA, PNN, QUEST, RBF, RELM, RF, ROC, SVM, and MDS.	Linear RBF	ANOVA	2,942	98.87	✗	✗
Sadiq et al. (21)	2021	SVM, GBM, and RF.	Linear	RF	5,066	93%	✗	✗
Uçucu and Azik (23)	2024	ANN and DT.	–	DT	396	99.54%	✗	✗
Tran et al. (39)	2023	KNN, SVM, RF, and MLP.	Linear	RF	1,555	98.45%.	✗ ✗	✗
Das et al. (40)	2020	DT, NB, and ANN.	RBF	DT, RF	1,076	93.835%	✗	✗
Zhang et al. (42)	2024	DT, LR, LSVC, RF, and SDG.	Linear	Correlation analysis, L1, deepwise, and beckman coulter DxAI platform	2,851	93.38%	✗	✗ ✗ ✗ ✗
Jahan et al. (43)	2021	ANN, C4.5 and NB.	–	–	3,947	88%	✗	✗

Equation 18 defines the RBF kernel.


$$k(p,y)=e\;(\frac{\|p-y\|2}{2\;\gamma^{2}})$$ (18)

Equation 19 shows the weight update procedure utilizing the RBF kernel.


$$W^{R}={\left[\begin{array}{ccc} W_{1x1}^{R} & W_{1x2}^{R} & \begin{array}{ccc} W_{1x3}^{R} & \cdots & W_{1\mathrm{xm}}^{R} \end{array}\\ W_{2x1}^{R} & W_{2x2}^{R} & \begin{array}{ccc} W_{2x3}^{R} & \ldots & W_{2\mathrm{xm}}^{R} \end{array}\\ \begin{array}{c} W_{3x1}^{R}\\ \vdots \\ W_{\mathrm{nx}1}^{R} \end{array} & \begin{array}{c} W_{3x2}^{R}\\ \vdots \\ W_{\mathrm{nx}2}^{R} \end{array} & \begin{array}{ccc} \begin{array}{c} W_{3x3}^{R}\\ \vdots \\ W_{\mathrm{nx}3}^{R} \end{array} & \begin{array}{c} \ldots \\ \ddots \\ \cdots \end{array} & \begin{array}{c} W_{3\mathrm{xm}}^{R}\\ \vdots \\ W_{\mathrm{nxm}}^{R} \end{array} \end{array} \end{array}\right]}_{\mathrm{NxM}}$$ (19)

##### 4.1.1.4 Deep kernel

DK techniques create adaptable similarity functions that are tailored for particular data tasks by combining kernel learning with deep neural network architectures. In order to extract representations before calculating similarity, learnable Deep Kernels map input features through hidden layers. A Deep Kernel can enhance classification in *β*-thalassemia carrier detection by automatically identifying the most important features and managing feature interactions more skillfully.

Equation 20 illustration the weight update procedure applying the DK kernel.


$$W^{\mathrm{DK}}={\left[\begin{array}{ccc} W_{1x1}^{\mathrm{DK}} & W_{1x2}^{\mathrm{DK}} & \begin{array}{ccc} W_{1x3}^{\mathrm{DK}} & \cdots & W_{1\mathrm{xm}}^{\mathrm{DK}} \end{array}\\ W_{2x1}^{\mathrm{DK}} & W_{2x2}^{\mathrm{DK}} & \begin{array}{ccc} W_{2x3}^{\mathrm{DK}} & \ldots & W_{2\mathrm{xm}}^{\mathrm{DK}} \end{array}\\ \begin{array}{c} W_{3x1}^{\mathrm{DK}}\\ \vdots \\ W_{\mathrm{nx}1}^{\mathrm{DK}} \end{array} & \begin{array}{c} W_{3x2}^{\mathrm{DK}}\\ \vdots \\ W_{\mathrm{nx}2}^{\mathrm{DK}} \end{array} & \begin{array}{ccc} \begin{array}{c} W_{3x3}^{\mathrm{DK}}\\ \vdots \\ W_{\mathrm{nx}3}^{\mathrm{DK}} \end{array} & \begin{array}{c} \ldots \\ \ddots \\ \cdots \end{array} & \begin{array}{c} W_{3\mathrm{xm}}^{\mathrm{DK}}\\ \vdots \\ W_{\mathrm{nxm}}^{\mathrm{DK}} \end{array} \end{array} \end{array}\right]}_{\mathrm{NxM}}$$ (20)

These results in Table 3 highlight not only the importance of choosing an appropriate kernel based on dataset characteristics but also underscore the critical impact of preprocessing steps such as standardization and class balancing in enhancing model robustness and generalization.

**Table 3:** Details of features.

Serial number	Attribute	Type	Range
1	Age	Numerical	0.0–1.0
2	Sex	Numerical	0.0–1.0
3	WBC	Numerical	1.1–49.2
4	RBC	Numerical	1.0–9.8
5	HGB	Numerical	1.3–2.7
6	HCT	Numerical	3.0–70.0
7	MCH	Numerical	12.0–42.0
8	MCV	Numerical	11.0–122.0
9	MCHC	Numerical	21.0–48.0
10	RWD	Numerical	9.0–17.0
11	PLT	Numerical	3.0–1087.0
12	Class	Numerical	0.0–1.0

The algorithms below show the description of training the models on the client side.

Algorithm 1 Client-side SVM training algorithm.
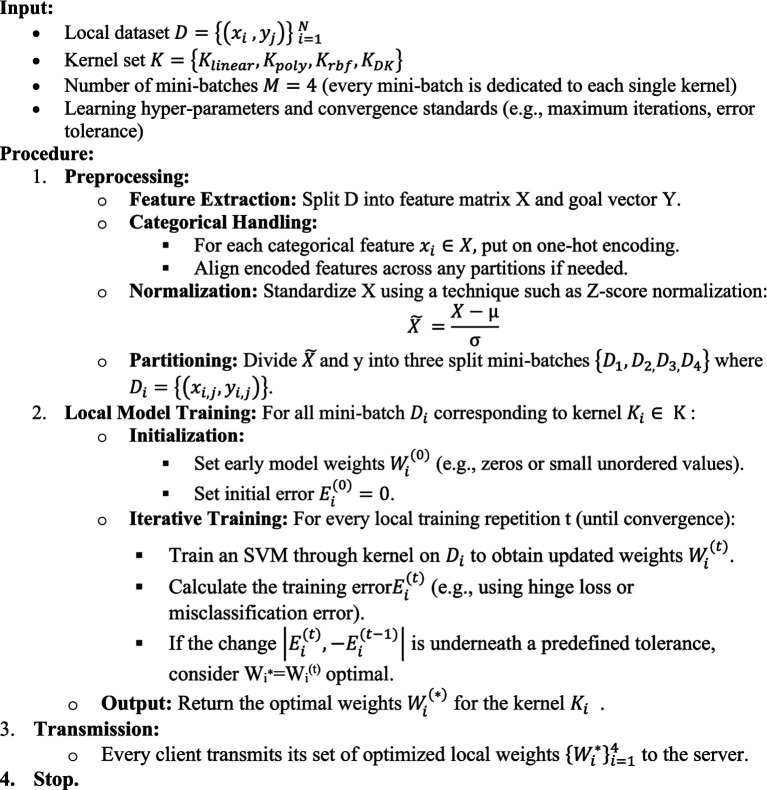

### 4.2 Federated learning framework: global models

In the final stage of our Experiment, the insights from the three SVM kernels are integrated within a federated learning framework, enabling multiple client models to train locally on their respective datasets while a global model consolidates their insights. In the proposed framework, the linear and RBF kernels function as client models, each specializing in capturing distinct data patterns. The linear kernel provides a clear and interpretable decision boundary, whereas the RBF kernel effectively models nonlinear relationships. Together, these models offer complementary perspectives on the classification task. The outputs and insights derived from these client models are subsequently aggregated into a global model, where the RBF kernel is employed, with its results presented in Table 4. This federated learning approach creates a strong and adaptable classification system by harnessing the strengths of individual client models and benefiting from the global model’s ability to aggregate insights.

Algorithm 2 Server-side federated algorithm.
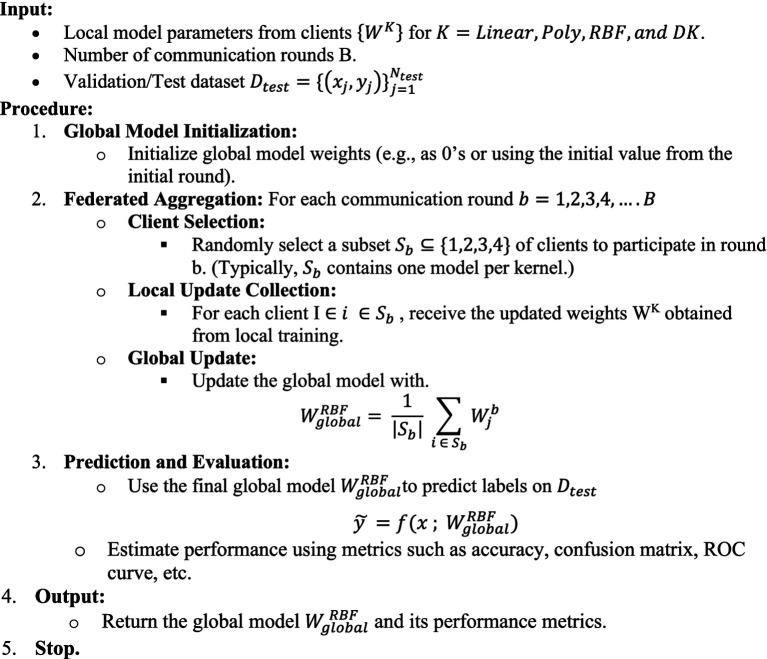

**Table 4:** Complete results of proposed model.

Kernal	Precision		Recall		F1 score		Acc	CV	Mean CV
	Macro avg	Weighted avg	Macro avg	Weighted avg	Macro avg	Weighted avg			
Linear	0.956	0.957	0.956	0.956	0.956	0.956	0.956	0.950, 0.948, 0.951, 0.943, 0.947	0.948
Poly	0.977	0.977	0.977	0.977	0.977	0.977	0.977	0.993, 0.983, 0.984, 0.991, 0.990	0.988
RBF	0.984	0.984	0.984	0.984	0.984	0.984	0.984	0.993, 0.989, 0.990, 0.992, 0.991	0.991
DK	0.932	0.932	0.932	0.932	0.932	0.932	0.932	0.959, 0.960, 0.962, 0.952, 0.955	0.942

## 5 Results and discussion

Computer Hardware: Intel Xeon CPU @ 2.20 GHz (virtualized), NVIDIA Tesla T4 GPU with 16 GB HBM2 memory, and 12.7 GB of system RAM. Operating System: Ubuntu 18.04 LTS (via Google Colab’s managed backend). Software Frameworks: Python 3.8, TensorFlow 2.13.0 for model implementation and training, scikit-learn 1.2 for preprocessing and SVM kernels, Pandas 1.5, and NumPy 1.24 for data manipulation.

We determined a number of evaluation metrics, including Precision, Recall, F1 Score, Accuracy, and Specificity, in order to thoroughly evaluate the performance of our model. Particularly when there is a class imbalance or variable costs of misclassification, these metrics offer a comprehensive picture of the model’s efficacy. These metrics have the following definitions and formulas:

The percentage of positive identifications that were truly accurate is known as precision (positive predictive value).


$$\text{Precision}=\frac{\text{True Positive}\;(\mathrm{TP})}{\text{True Positive}\;(\mathrm{TP})+\text{FalsePositive}\;(\mathrm{FP})}$$

Recall (also known as sensitivity or true positive rate): Shows the percentage of real positives that the model correctly detected.


$$\text{Recall}=\frac{\text{True Positive}\;(\mathrm{TP})}{\text{True Positive}\;(\mathrm{TP})+\text{False Negative}\;(\mathrm{FN})}$$

F1 Score: A balance between precision and recall, calculated as the harmonic mean of the two metrics. When there is an unequal distribution of classes, it is especially helpful.


$$F1\;\text{Score}=2\;x\frac{\text{Precision}\;x\;\text{Recall}}{\text{Precision}+\text{Recall}}$$

Accuracy: By calculating the percentage of true results both true positives and true negatives—among all the cases analyzed, accuracy indicates the model’s overall correctness.


$$\text{Accuracy}=\frac{\text{True Positive}\;(\mathrm{TP})+\text{True Negative}\;(\mathrm{TN})}{\text{Total Parameters}\;(\mathrm{TP}+\mathrm{TN}+\mathrm{FP}+\mathrm{FN})}$$

Specificity: The percentage of true negatives that were accurately identified is known as specificity (or true negative rate). In situations where false positives are especially undesirable, it is essential.


$$\text{Specificity}=\frac{\text{True Negatives}\;(\mathrm{TN})}{\text{True Negatives}\;(\mathrm{TN})+\text{FalsePositives}\;(\mathrm{FP})}$$

We hope to ensure the dependability and efficacy of our model in real-world applications by using these metrics to provide an open and comprehensive assessment of its performance.

At the client side, the kernels of the RBF variant achieved the highest test accuracy of 0.99%, followed closely by the polynomial kernel at 0.98 and the linear kernel at 0.95. The federated ensemble SVM framework produced exceptional classification performance across all kernels. These findings were validated by cross-validation, which revealed strong generalization under 5-fold splits with mean accuracies of 0.991 for RBF, 0.988 for polynomial, 0.948 for linear kernels, and 0.961 for the DK. Accuracy was matched by precision, recall, and F1-scores, all of which exceeded 98.4% for nonlinear kernels and showed balanced sensitivity and specificity, important for medical diagnostics. By combining these local kernels, the global federated model obtained a consolidated accuracy of 98.4%, confirming that federated averaging maintains privacy without sacrificing predictive ability. Near-perfect class separation and little overlap between carrier and non-carrier distributions were highlighted by ROC AUC values of 1.00 for polynomial and RBF kernels and 0.98 for linear. The clinical reliability of the model in identifying *β*-thalassemia carriers from CBC indices was confirmed by confusion matrices, which showed less than 1% misclassification errors. All things considered, these findings demonstrate how well multi-kernel SVMs work in a federated environment, providing a high-accuracy, privacy-preserving option for extensive carrier screening. Table 5 shows the detailed results of client-side kernels.

**Table 5:** Detailed results of the client side (kernels).

Kernal	Precision		Recall		F1 score		Acc	CV	Mean CV
	Macro avg	Weighted avg	Macro avg	Weighted avg	Macro avg	Weighted avg			
Linear	0.989	0.989	0.989	0.988	0.989	0.989	0.957	0.950, 0.948, 0.951, 0.943, 0.946	0.948
Polynomial	0.985	0.985	0.985	0.985	0.985	0.985	0.985	0.993, 0.983, 0.984, 0.991, 0.990	0.988
RBF	0.989	0.989	0.989	0.989	0.989	0.989	0.989	0.993, 0.989, 0.990, 0.992, 0.991	0.991
DK	0.961	0.961	0.961	0.961	0.961	0.961	0.961	0.959, 0.960, 0.962, 0.952, 0.955	0.958

### 5.1 Results of client-side kernels

**Figure 4:**
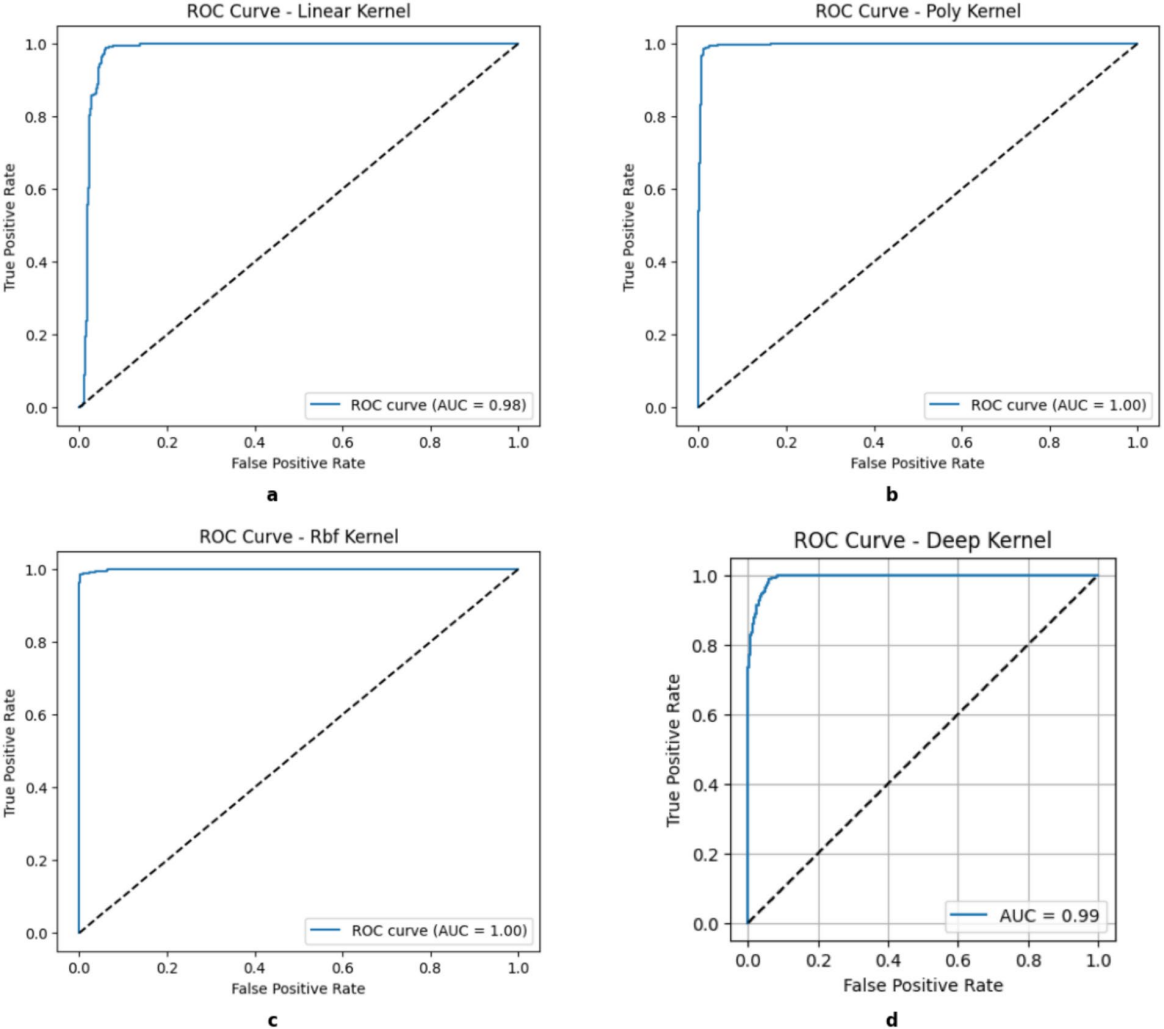
ROC curves of kernels. **(a)** Linear, **(b)** Poly, **(c)** RBF, **(d)** DK.

The in-depth comparison of the classification performance and generalization ability of the three SVM and DK, i.e., Linear, Polynomial, RBF, and DK, is depicted by the Receiver Operating Characteristic (ROC) curve analysis of all kernels such as Figure 4 that the Linear kernel has an Area Under Curve (AUC) rate of 0.98 and DK achieved the 0.98, demonstrating its efficient classification. However, the other nonlinear kernels surpass it to indicate that it struggles with complex and high-dimensional feature relationship that leads to inefficiencies in minor classifications. The Polynomial and RBF Kernels achieved an AUC of 1.00 and indicated that no misclassification errors were observed, thereby showcasing their potential in modeling nonlinear relationships. This near-perfect classification makes them ideal for datasets with intricate class separations. Therefore, the results of these area under curve values show the potential of these nonlinear kernels with superior classification reliability, but they are limited to dataset complexity and the optimal generalizability of the data, as there is a need for balanced bias.

**Figure 5:**
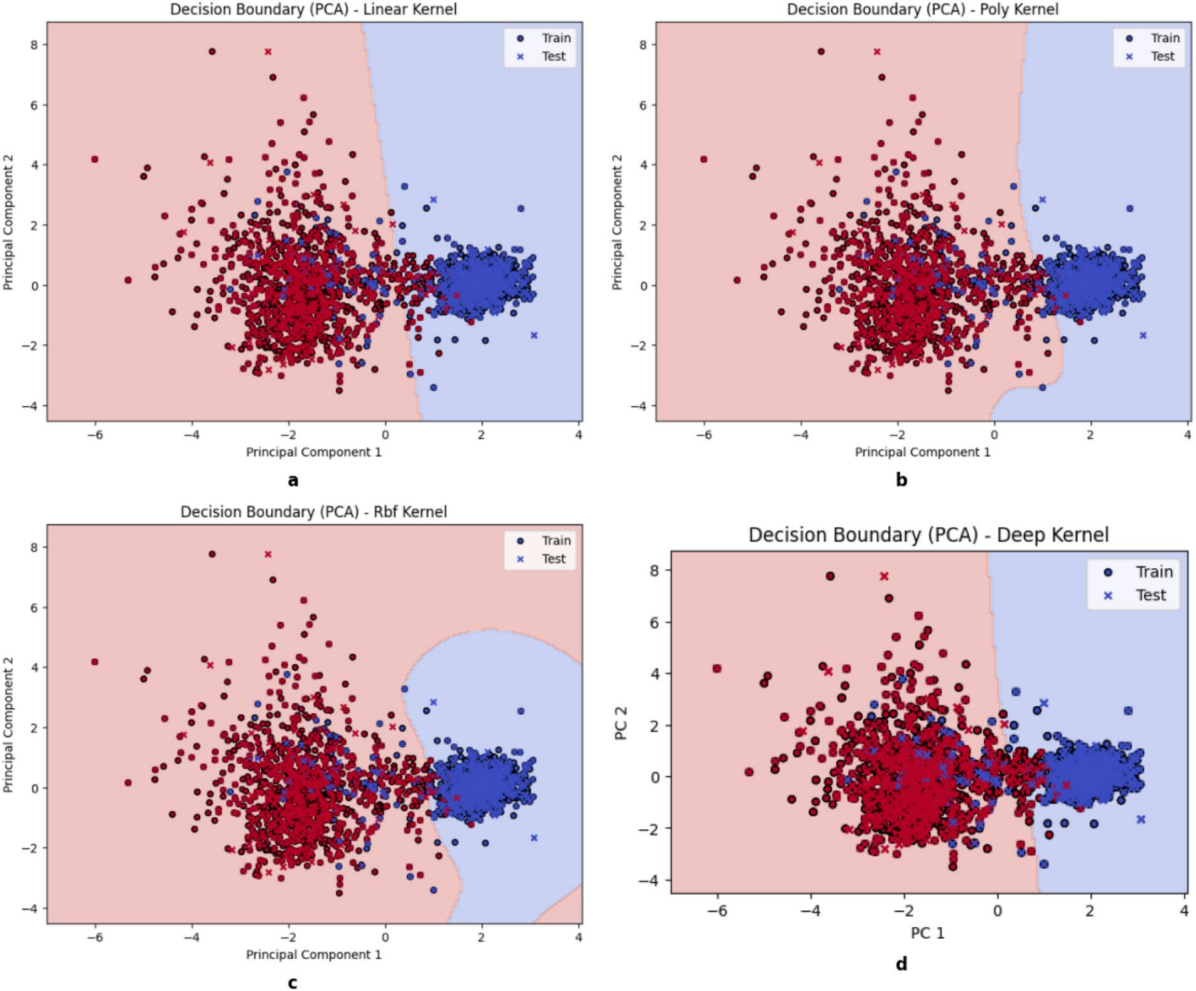
Decision boundary of kernels. **(a)** Linear, **(b)** Poly, **(c)** RBF, **(d)** DK.

Figure 5 illustrates the projection of different kernel classification decision boundaries onto a two-dimensional space using principal component analysis (PCA). The filled circles (●) represent the training data used to train the model, and the crosses (×) represent the test data used specifically to test the model. The Support Vector Machines (SVMs) work by utilizing various kernel functions to shape decision boundaries that can influence model adaptability, performance, and generalization. A linear kernel forms a straightforward boundary to make it computationally efficient with linearly separable data, but it still struggles with more intricate patterns. The polynomial kernel introduces curvature that allows it to handle moderate non-linearity, but it is also limited to require careful selection of its degree for the prevention of overfitting. By learning task-specific feature representations and capturing intricate interactions, deep kernels enhance tabular data classification and are useful for *β*-thalassemia carrier detection. However, scalability on large datasets may be limited due to the high computational resources and careful tuning required.

Moreover, the Radial Basis Function (RBF) kernel maps data in a space with higher dimensions to create highly flexible boundaries for capturing complex relationships effectively. RBF is often the most accurate for complex datasets as it requires meticulous hyperparameter tuning and greater computational resources. A solid understanding of these kernels can help in choosing the best approach for optimal classification and model generalization.

**Figure 6:**
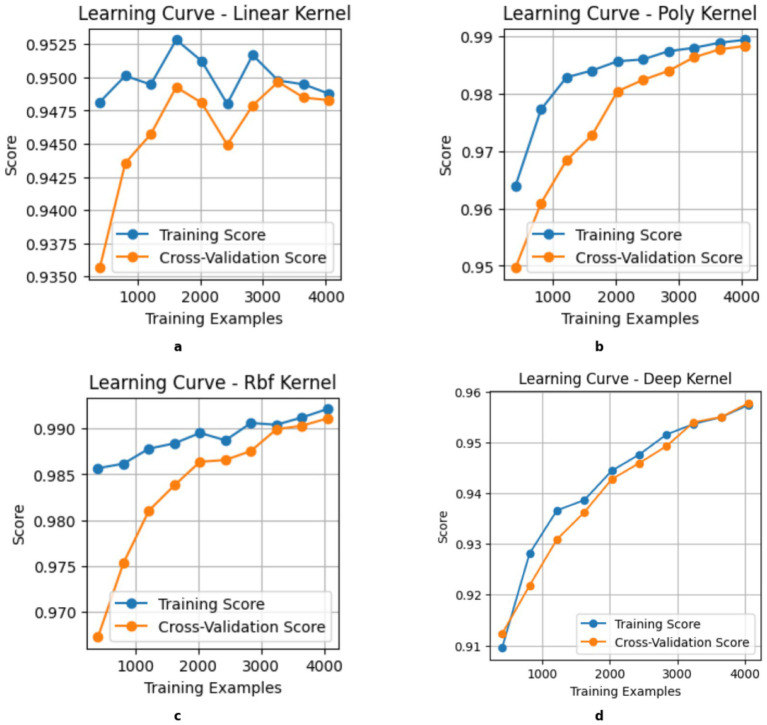
Learning Curve of kernels. **(a)** Linear, **(b)** Poly, **(c)** RBF, **(d)** DK.

The learning curve in Figure 6 shows that the x-axis represents the size of the training data, and the y-axis represents the classification performance metric. The solid blue line labeled “Training Score” shows the model’s performance on the training dataset, and the solid orange line labeled “Cross-Validation Score” represents the model’s average performance on unknown data and serves as a measure of its generalizability. A small difference between the two curves indicates good generalizability and low overfitting, while a large difference indicates high model variance. The Linear Kernel (a) shows noticeable fluctuations in both training and validation scores and suggests moderate variance as it is sensitive to data changes. Although its performance improves with more data, the inconsistency in validation results indicates that it struggles with capturing complex patterns. The Polynomial Kernel (b) and Deep Kernel (d) exhibits a steady improvement with training and validation scores that gradually align with large datasets. This attribute shows increased model stability and better classification accuracy. The RBF (c) Kernel stands out as the most effective in achieving the highest accuracy to display a smooth learning curve. The small gap between training and validation scores proves its generalizability with less overfitting.

**Figure 7:**
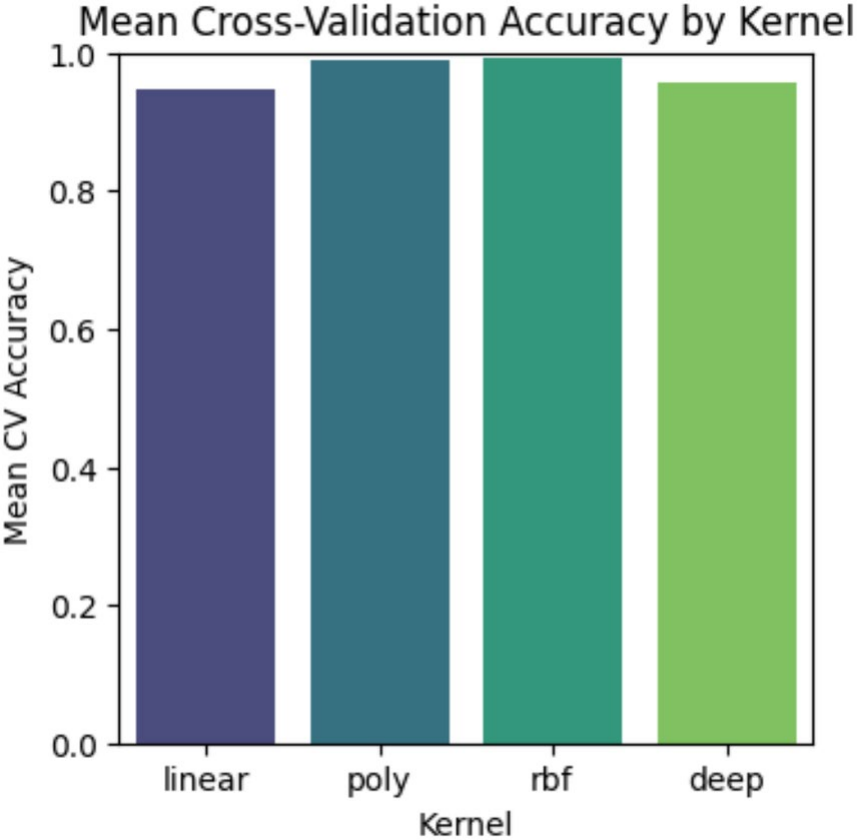
Validation between kernels.

Comparing in Figure 7, the Mean Cross-Validation Accuracy of different SVM and Deep kernels helps in understanding how they perform across various decision boundaries. The bar chart shows that all three kernels achieve strong accuracy; the RBF kernel consistently performs the best among all the kernels. As the higher accuracy often indicates better performance, it is also important to consider its limitations, i.e., the risk of overfitting with more complex kernels. Thus, choosing the right kernel requires balancing accuracy and generalization based on data characteristics.

**Figure 8:**
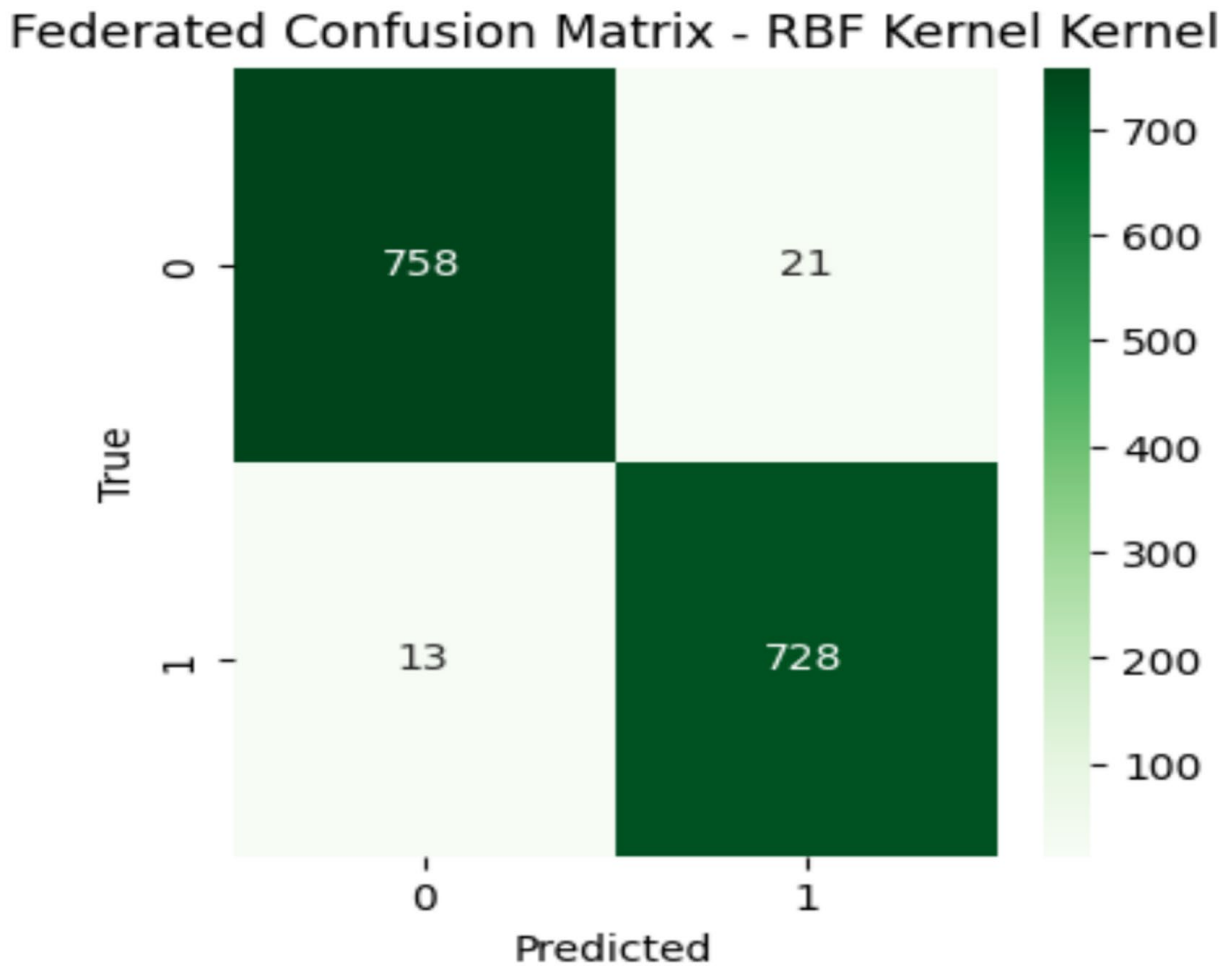
Confusion matrix of global model.

Table 4 shows the results of the proposed client-side models. It is clearly shown that RBF achieved the maximum accuracy, precision, recall, F1 Score, and specificity as compared to the Linear and Poly kernels. Both the accuracy value of 0.984 and the specificity value of 0.989 show the model’s performance efficiency. This federated learning setup comes with several key benefits, i.e., it uses distributed learning that allows local models to focus on specific patterns for their data, which is especially useful for diverse datasets. Moreover, the RBF kernel acts as a global average that effectively combines decision boundaries from the linear and Poly kernels. The confusion matrix confirms that strong performance leads to better classification accuracy and generalization, as depicted in Figure 8. At last, this approach enhances both data privacy and scalability that enabling models to train on local data without sharing raw information with a central server.

### 5.2 Results of global model

Table 6 shows the low results proposed federated learning model with two different agnostic models, i.e., Artificial Neural Network (ANN), three different activation functions Linear, Poly, and RBF, along with Kernel Logistic Regression (KLR), has also been trained with the proposed model and the results are shown.

**Table 6:** Comparison with agnostic models.

Kernels	Artificial neural network (ANN)						
	Precision	recall	F1 score	accuracy	specificity	Mean CV	FL
Linear	0.94	0.96	0.95	0.95	0.93	0.93	0.94
Poly	0.95	0.97	0.96	0.96	0.95	0.95	0.95
RBF	0.96	0.97	0.96	0.96	0.95	0.96	0.95
Kernel logistic regression (KLR)							
Linear	0.88	0.89	0.89	0.82	0.89	0.89	0.79
Poly	0.90	0.90	0.90	0.88	0.89	0.89	0.86
RBF	0.92	0.92	0.92	0.93	0.93	0.92	0.92

### 5.3 Explainable AI

SHAP analysis was applied to the baseline RBF-kernel SVM to quantify the contribution of each input variable to the predicted class probabilities. The resulting importance profiles identify hemoglobin, RBC count, MCV, RDI, and hematocrit as the dominant determinants of classification, with higher values increasing the likelihood of the positive target class and lower values favoring the alternative class. These methods offer reliable ways to explain AI decisions at both local and global levels with no modifications in the model (63–65). This study incorporates advanced explainable AI techniques for the improvement in the interpretability of the SVM model with an RBF kernel.

**Figure 9:**
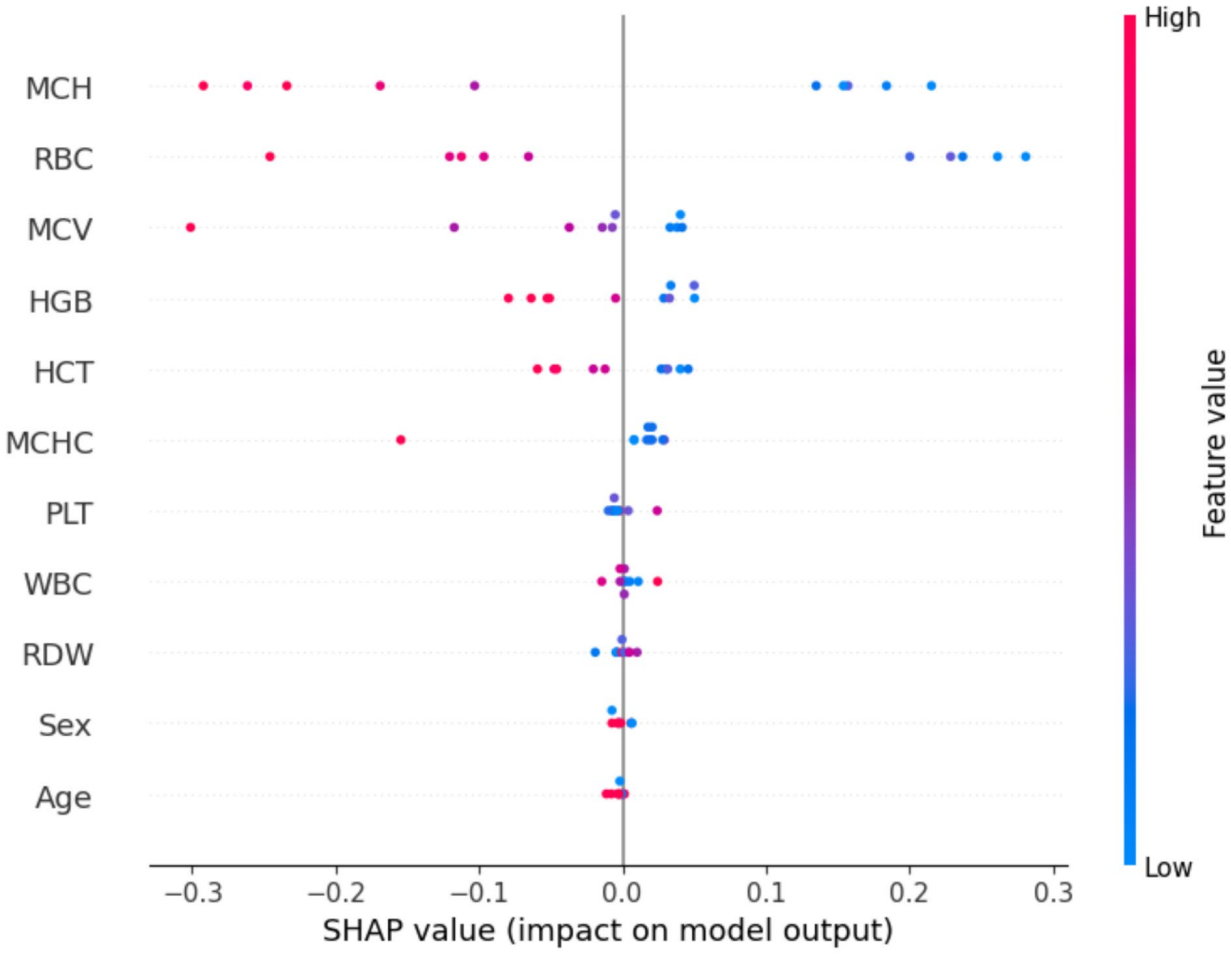
Shape value of the dataset.

In Figure 9, the SHAP summary plot, features on the y-axis are ordered by their mean absolute SHAP value, while the x-axis encodes the SHAP value, with positive (right) and negative (left) contributions, respectively, increasing or decreasing the predicted probability of the target class; points are color-mapped from blue (low feature values) to red (high feature values), jointly conveying the magnitude and direction of each feature’s effect on the model output.

**Figure 10:**
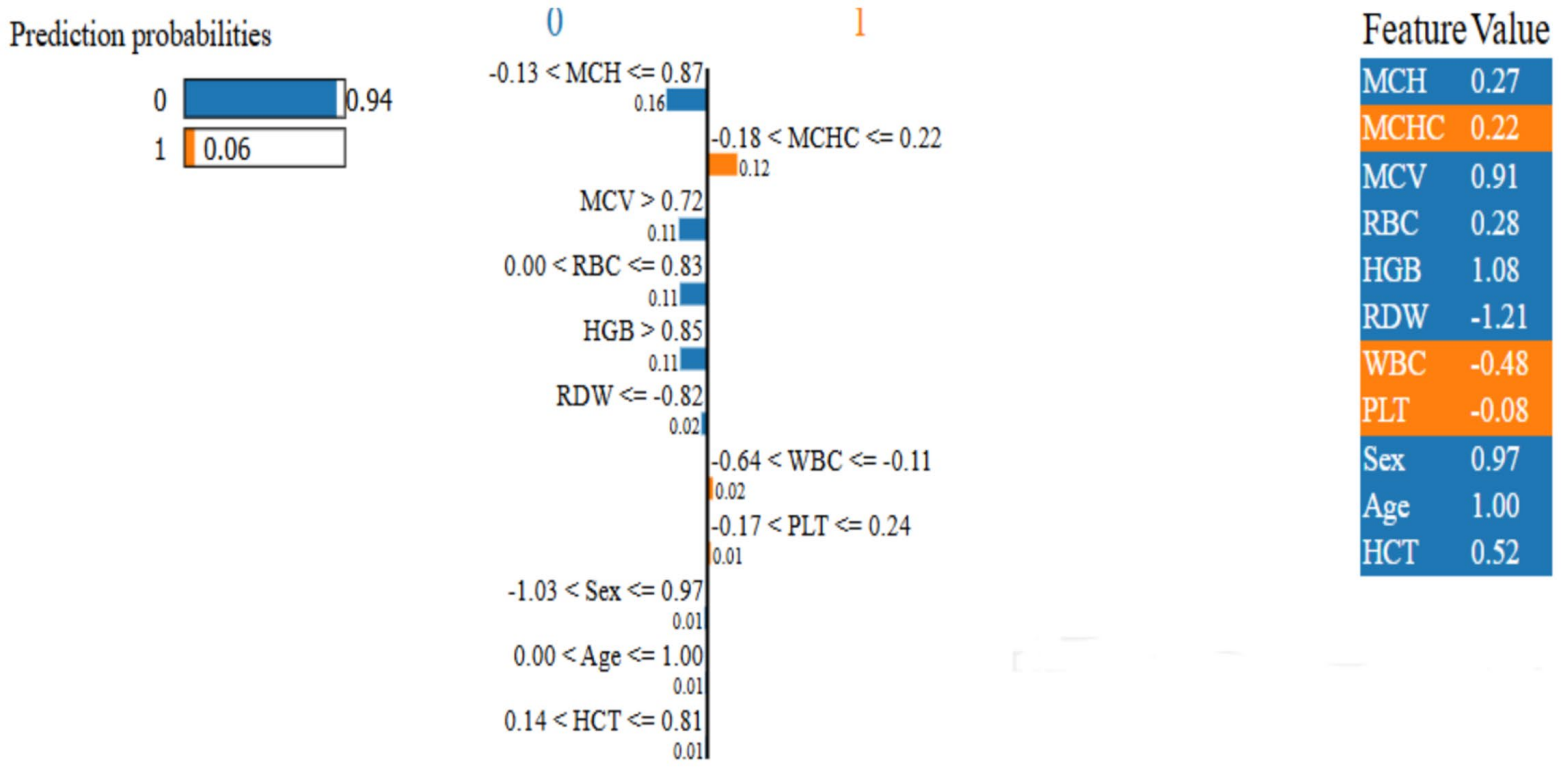
Proposed model local interpretable model-agnostic explanations.

Figure 10, LIME was applied to the RBF-SVM to obtain instance-specific local surrogate models, revealing that red blood cell indices (MCV, RDI, Hb, RBC) consistently exert the strongest positive contributions to individual classifications, whereas PLT and WBC frequently attenuate the predicted probability, acting as negative or moderating factors. In the illustrated example, the explanation bar plot shows a high predicted probability for class 0 (0.94 vs. 0.06), with left-extending bars decreasing, and right-extending bars increasing, the likelihood of class 1 in proportion to their length. The feature importance ranking confirms the model prioritizes relevant hematological parameters, validating its alignment with medical expertise (44).

By rigorously optimizing our global model through techniques like RandomizedSearchCV and validating it on a comprehensive dataset of 5,066 patients, we achieved an outstanding accuracy of 98.4% (see Table 7). Additional performance metrics further highlight the model’s reliability. ROC curves with AUC values consistently exceeding 0.98, with detailed confusion matrices and error histograms, indicate a well-balanced sensitivity and specificity. Visual tools, i.e., PCA plots, depict distinct decision boundaries, and learning curves confirm stable convergence with minimal overfitting risk. Furthermore, combining outputs from multiple kernels through majority voting significantly improved the model’s ability to generalize across diverse datasets.

**Table 7:** Performance comparisons of proposed model with previous work.

Index No.	Evaluation				FL	XAI	Dataset used
	Accuracy	F1 score	Precision	Recall			
AlAgha et al. (15)	99.5%	–	–	–	No	No	Identifying β-thalassemia carriers (Palestine Avenir Foundation, Gaza Strip)
Das et al. (20)	99.21%	98.84%	99.30%	–	No	No	β-thalassemia trait antenatal females were collected from the Department of Hematology at PGIMER, Chandigarh, India
Sadiq et al. (21)	93%	90%	89%	89%	No	No	β-thalassemia carrier from red blood cell
Tran et al. (39)	98.45%	97.65	94.67%	99.23%	No	No	Thalassemia screening of pregnant women and their husbands, Vietnam National Hospital
Das et al. (40)	95.27%	–	–	–	No	No	Beta thalassemia and HbE carrier screening, Chandigarh India
Zhang et al. (42)	93.38%	90.91%	–	92.59%	No	No	Thalassemia trait (TT) gene carriers in a non-anemic population, Sun Yat-sen University
Jahan et al. (43)	88.56%	–	–	–	No	No	β-thalassemia traits (BTT) samples of pregnant women who underwent thalassemia screening, tertiary care hospital
Proposed	98.4%	98.4%	98.4%	98.4%	YES	YES	β-thalassemia carrier

In Table 7. The performance of models used in (15, 20) in terms of accuracy is higher than our proposed model, but they did not undergo federated learning, and also did not apply interpretable artificial intelligence. In AlAgha et al. (15), the dataset used is obtained from the Gaza Strip, Palestine. Whereas the dataset used in Das et al. (20) is the antenatal dataset that is gathered from pregnant ladies. In a decentralized federated learning approach, there might be some loss due to the transfer of only the model parameters from the client side to the server node, and there will be no biased approach in federated learning, but in a traditional machine learning model, compromises a biasness. So, the novelty of the proposed work is the use of the SVM algorithm in federated learning for the prediction of *β*-Thalassemia Carrier, and different kernels of SVM have been used in the client side for model training.

## 6 Conclusion

In this work, we showed that a federated SVM and DK can accurately screen for β-thalassemia carriers using complete blood count (CBC) data. By combining linear, polynomial, and RBF and DK, our model captured complex patterns in red-cell indices (MCV, MCH, RBC count, RDW, etc.) and achieved a classification accuracy of 98.4%, on par with the best prior SVM-based formulas (which reported 98.4% Accuracy). Importantly, training was done in a federated manner, meaning that only model parameters were exchanged, protecting patient privacy. We also used explainable Using LIME and SHAP, feature importance analyses demonstrate that the model relies heavily on blood indices that are already established as differentiating carriers from non-carriers.

Further research should focus on increasing the model’s sensitivity with additional biomarkers, validating performance across diverse areas in the biomedical industry, exploring other federated learning types for robustness, and conducting clinical deployment studies to measure usability and trust. Federated learning technique, based on Visual data, will be developed to help medical practitioners review particular cases with the same latest prescription with the help of a global model generated by the FL.

While promising, the study has limitations that the dataset used in this study is region-specific and primarily represents the population of Pakistan; this may limit the model’s ability to be applied to larger, more diverse populations. The safe transfer of encrypted trained models from client devices to the central server presents difficulties when federated learning is put into practice. To increase the suggested model’s generalizability and robustness across a range of demographics, we plan to expand the dataset in subsequent research by adding multi-regional and multi-ethnic samples. To further ensure more secure and effective model updates between clients and the central server, we also intend to integrate sophisticated privacy-preserving techniques like differential privacy, secure aggregation, and homomorphic encryption into the federated learning framework.

## Data Availability

The original contributions presented in the study are included in the article/supplementary material, further inquiries can be directed to the corresponding author.
